# How the clinical research community responded to the COVID-19 pandemic: an analysis of the COVID-19 clinical studies in ClinicalTrials.gov

**DOI:** 10.1093/jamiaopen/ooab032

**Published:** 2021-04-20

**Authors:** Zhe He, Arslan Erdengasileng, Xiao Luo, Aiwen Xing, Neil Charness, Jiang Bian

**Affiliations:** 1 School of Information, Florida State University, Tallahassee, Florida, USA; 2 Department of Statistics, Florida State University, Tallahassee, Florida, USA; 3 Department of Computer Information and Graphics Technology, Indiana University–Purdue University Indianapolis, Indianapolis, Indiana, USA; 4 Department of Psychology, Florida State University, Tallahassee, Florida, USA; 5 Department of Health Outcomes and Biomedical Informatics, University of Florida, Gainesville, Florida, USA

**Keywords:** COVID-19, clinical trial, eligibility criteria, natural language processing

## Abstract

**Objective:**

In the past few months, a large number of clinical studies on the novel coronavirus disease (COVID-19) have been initiated worldwide to find effective therapeutics, vaccines, and preventive strategies for COVID-19. In this study, we aim to understand the landscape of COVID-19 clinical research and identify the issues that may cause recruitment difficulty or reduce study generalizability.

**Methods:**

We analyzed 3765 COVID-19 studies registered in the largest public registry—ClinicalTrials.gov, leveraging natural language processing (NLP) and using descriptive, association, and clustering analyses. We first characterized COVID-19 studies by study features such as phase and tested intervention. We then took a deep dive and analyzed their eligibility criteria to understand whether these studies: (1) considered the reported underlying health conditions that may lead to severe illnesses, and (2) excluded older adults, either explicitly or implicitly, which may reduce the generalizability of these studies to the older adults population.

**Results:**

Our analysis included 2295 interventional studies and 1470 observational studies. Most trials did not explicitly exclude older adults with common chronic conditions. However, known risk factors such as diabetes and hypertension were considered by less than 5% of trials based on their trial description. Pregnant women were excluded by 34.9% of the studies.

**Conclusions:**

Most COVID-19 clinical studies included both genders and older adults. However, risk factors such as diabetes, hypertension, and pregnancy were under-represented, likely skewing the population that was sampled. A careful examination of existing COVID-19 studies can inform future COVID-19 trial design towards balanced internal validity and generalizability.

LAY SUMMARY

Since the outbreak of COVID-19 in early 2020, thousands of clinical studies have been conducted to evaluate the efficacy and safety of various types of treatments and vaccines in human. COVID-19 clinical studies play a crucial role in controlling the virus. Yet it is unclear what types of patients were considered by these studies. This study analyzed 3765 COVID-19 clinical study summaries downloaded from a major clinical trial registry ClinicalTrials.gov. We employed natural language processing (NLP) techniques to parse the study description and eligibility criteria of these studies and then performed descriptive and clustering analysis on the parsing results. We found that older adults were not systematically excluded but pregnant women were often excluded. It was also found that the known risk factors such as diabetes, hypertension, obesity, and asthma, which may lead to serious illnesses, were considered by less than 5% of the studies according to their study description and eligibility criteria. This study provides an evidence that NLP can be applied to examine the design of clinical studies and identify issues that may cause delays in patient recruitment and the lack of real-world population representativeness.

## INTRODUCTION

The severe acute respiratory syndrome coronavirus 2 (SARS-CoV-2) and the associated coronavirus disease (COVID-19) broke out in December 2019 and has quickly become a global pandemic with serious health and social consequences.^1^ As of February 11, 2021, more than 107 million confirmed cases have been reported around the world and about one-fourth are from the United States.^2^ Globally, more than 2 million people have died due to COVID-19 and 472 000 in the United States alone. In April 2020, the National Institutes of Health (NIH) launched the Accelerating COVID-19 Therapeutic Interventions and Vaccines (ACTIV) public–private partnership to prioritize and speed up the clinical evaluation of the most promising treatments and vaccines.^3^ In July 2020, NIH released its strategic plan for COVID-19 research to speed up the development of treatments, vaccines, and diagnostics.^4^ Traditionally, it may take years to discover, develop, and evaluate a therapeutic agent; nevertheless, for COVID-19, the goal has been to compress the timeline to months while continuing to apply rigorous standards to ensure safety and efficacy. Strategies such as applying complex computer-generated models of SARS-Cov-2 and its biological processes to determine key interactions and pathways have been applied to develop therapeutic agents and vaccines (eg, monoclonal antibodies) to neutralize the virus. Significant efforts have also been made to screen existing drugs approved for other indications to treat COVID-19.^4^ Recently, the COVID-19 vaccines developed and manufactured by Pfizer-BioNTech and Moderna have been approved to be administered in a few countries including the United Kingdom,^5^ Canada,^6^ and the United States.^7^

Clinical studies, especially randomized controlled trials (RCT), are the gold standard for evaluating the efficacy and safety of a treatment. Regardless of the techniques (eg, in vivo, in silico, or in vitro) used for drug discovery and development, the therapeutics and vaccines have to go through 3 phases of clinical trials to evaluate their efficacy and safety before approvals (eg, by the US Food and Drug Administration [FDA]) can be granted for mass production and use in the general population. In the past few months, many COVID-19 clinical studies have been launched around the world, leading to situations where studies have to compete for participants from the same pool of eligible participants. Trials, such as those for the promising drug—*Remdesivir—*were suspended due to the lack of trial participants in China.^8^ Other issues, such as population representativeness, are also critical. In the past, older adults are often excluded from clinical trials with overly restrictive exclusion criteria, which lead to concerns on the generalizability of those clinical studies across many disease domains.^9^ A recent New York Times article conjectured that older adults are left out from COVID-19 trials.^10^ It is therefore important to understand the landscape of COVID-19 clinical research and further identify the gaps and issues that may cause delays in patient recruitment and the lack of real-world population representativeness, especially for older adults.

To date, 12 other studies have analyzed registered COVID-19 clinical studies^11–22^ (see Supplementary Material I for details). For example, Wang et al^11^ analyzed the basic characteristics and the drug interventions of 306 COVID-19 trials from ClinicalTrials.gov as of April 3, 2020. Pundi et al^12^ analyzed 1551 COVID-19 studies registered between March 1, 2020 and May 19, 2020 in ClinicalTrials.gov and focused on basic characteristics, primary and secondary outcomes, and study design. Kim et al^22^ evaluated the impact of frequently used quantitative eligibility criteria in 288 COVID-19 studies (as of June 18, 2020) on the recruitment and clinical outcomes using the EHR data of COVID-19 patients in Columbia University Irving Medical Center. While most of these studies analyzed the basic characteristics of the included trials, some studies further analyzed the interventions,^18–21^ locations,^13^^,^^14^^,^^18^ data monitoring characteristics,^14^ timing of the registration and enrollment,^15^^,^^21^ outcomes,^19^^,^^20^ risk of biases,^16^ Medical Subject Heading (MeSH) keywords (Words or phrases that best describe the protocol),^19^ sample size,^17^ and a subset of eligibility criteria.^16^^,^^22^ Nonetheless, existing studies have not comprehensively investigated the categorical eligibility criteria and the consideration of known risk factors of severe illnesses in COVID-19 clinical studies. To do so, advanced approaches such as natural language processing (NLP) are necessary. As such, we can gain a better understanding of the landscape of COVID-19 research and answer a number of important research questions: (1) What eligibility criteria are used in COVID-19 clinical studies? (2) Further, as more COVID-19 cases have been identified and treated over time, we have accumulated important knowledge on the underlying health conditions and other risk factors that may cause severe illness among COVID-19 patients (eg, hypertension and diabetes).^23^ Have existing clinical studies considered these known risk factors? (3) Last but not the least, because of the concerns on study generalizability in older adults, it is of interest to assess whether the COVID-19 clinical studies excluded subjects with common chronic conditions that are prevalent in older adults, potentially leading to their underrepresentation.

In this study, we conducted a systematic analysis of the registered clinical studies on COVID-19 (as of November 27, 2020) from ClinicalTrials.gov to answer the aforementioned research questions. The contribution of this article is multi-fold: (1) it systematically summarizes various important aspects of the COVID-19 clinical studies; (2) it identifies the research gaps on the risk factors related to serious illness caused by COVID-19; (3) it groups COVID-19 studies based on their eligibility criteria, and (4) it identifies salient exclusion criteria that may implicitly exclude older adults, who are most vulnerable and should be studied when evaluating the efficacy and safety of COVID-19 treatments and vaccines. Our findings could inform future trials designed for COVID-19 treatment and prevention and identify strategies to rapidly but appropriately stand up a large number of clinical studies for future pandemics similar to COVID-19.

## MATERIALS AND METHODS

### Data source

ClinicalTrials.gov, built and maintained by the US National Library of Medicine, is the largest clinical study registry in the world.^24^ In the United States, all drugs and devices regulated by the US Food and Drug Administration (FDA) are required to be registered on the ClinicalTrials.gov. ClinicalTrials.gov is thus considered as the most comprehensive trial registry in the world and has been widely used for secondary analysis.^25^

### Dataset acquisition and processing

From ClinicalTrials.gov, we downloaded records of 4028 clinical studies that are tagged with a condition of “COVID-19” or “SARS-CoV-2” on November 27, 2020. We excluded studies tagged with the study type “Expanded Access” and studies that were tagged as patient registries, leaving 3765 records that met our inclusion criteria. The reason for excluding “Expanded Access” studies is that the original studies have been included. The reason for excluding patient registries is that they do not use selective eligibility criteria and are probably biased in different ways than clinical trials. We extracted the NCTID (an unique identifier of a study record), conditions, agency, agency class, brief summary, detailed summary, status, start date, eligibility criteria, enrollment, study phase, study type, intervention type, intervention name, study design (ie, allocation, masking, observation model, time perspective), primary purpose, and endpoint classification. We split the eligibility criteria into inclusion criteria and exclusion criteria and further extracted individual criteria for NLP. To identify the top frequently tested drugs, we extracted the drugs information from the “intervention” field from the study record. We used QuickUMLS to normalize the drug names and removed the dosage information before analyzing their frequencies.

### Consideration of risk factors in COVID-19 clinical studies

We first identified known risk factors of COVID-19 from online resources such as the Centers for Disease Control and Prevention (CDC)^26^ and Mayo Clinic^27^ (as of July 17, 2020). Then, we coded the risk factors with the concepts from Unified Medical Language System (UMLS). To do so, we used the risk factor terms as the input and identified their corresponding Concept Unique Identifiers (CUIs) using QuickUMLS^28^ with the default setting (Jaccard similarity threshold > 0.8, all semantic types included). As a concept of the UMLS is associated with its synonyms from UMLS source ontologies, we were able to unify all the terms mentioned in the text. Table 1 lists the risk factors that may lead to severe COVID-19 and their associated UMLS CUIs. This list was used as a dictionary in QuickUMLS^28^ to identify risk factors from the study description. As reported in Soldaini et al^28^, QuickUMLS achieved better performance than MetaMap and cTAKES on a number of benchmark corpora. Nevertheless, we manually reviewed the risk factors extracted by QuickUMLS on a random sample of 100 clinical studies; and QuickUMLS achieved a precision of 91% (We did not evaluate the recall due to lack of a benchmark dataset for trial description with all the risk factors annotated manually.). We also identified the studies that used these risk factors in the inclusion/exclusion criteria using the parsing results of the eligibility criteria parsing tool^29^ described below. *t*-test and analysis of variance (ANOVA) were employed to assess the association between the number of risk factors in the trial descriptions with the study type, intervention type, and primary purpose.

**Table 1.** UMLS CUIs for the risk factors for severe illness among COVID-19 patients reported by CDC and Mayo Clinic websites

Risk factors	UMLS CUIs
Old age	C0231337, C1999167
Males	C0086582
Chronic kidney disease	C1561643, C4075517, C4553188, C4075526
COPD	C0024117
Lung cancer	C0684249, C0242379, C1306460
Weakened immune system from solid organ transplant (weakened immune system 1)	C0029216, C0524930
Obesity	C0028754, C1963185
Serious heart conditions, such as heart failure, coronary artery disease, or cardiomyopathies	C0018802, C4554158, C0018801, C0010054, C1956346, C0878544, C0796094, C0020542
Sickle cell disease	C0002895
Asthma	C0004096
Neurological conditions, such as dementia	C0002395, C0011265, C0497327, C0014544, C0026769, C0455388, C1417325, C0030567, C0036572
Cerebrovascular disease (affects blood vessels and blood supply to the brain) such as stroke	C0678234, C1961121, C0549207, C1261287, C1522213, C0524466, C0038454, C0007282, C0595850, C0158570, C0002940
Cystic fibrosis	C0010674
Hypertension	C0020538, C1963138
Weakened immune system from blood or bone marrow transplant, immune deficiencies, HIV, use of corticosteroids, or use of other immune weakening medicines (weakened immune system 2)	C0005961, C3540726, C3540727, C0021051, C0279026, C3539185, C3540725, C0001617, C1955133
Pregnancy	C0032961
Liver diseases	C0023895
Pulmonary fibrosis (having damaged or scarred lung tissues)	C4553408, C0034069
Smoking	C1881674, C1548578, C0037369, C0453996
Diabetes	C0011847, C0011849
Thalassemia	C0039730, C0002312

### Analysis of eligibility criteria

#### Quantitative criteria

We used the open-source Valx tool^30^ to extract and standardize the quantitative eligibility criteria from the COVID-19 studies. Valx is a system that can extract numeric expressions from free-text eligibility criteria and standardize them into a structured format. For example, from the inclusion criterion “BMI > 25 kg/m^2^”, the variable name “BMI”, the comparison operator “>”, the threshold value “25”, and the measurement unit “kg/m^2^” were extracted into 4 discrete fields. Valx is also able to recognize synonyms of a variable and convert the units to standard ones. According to Hao et al,^30^ the F-scores for structuring HbA1c and glucose comparison statements for Type 2 diabetes trials are 97.8% and 92.3%, respectively. We then analyzed the frequency of the quantitative criteria and the threshold values used for patient eligibility determination.

#### Categorical eligibility features

To extract the categorical eligibility features from COVID-19 studies, we used a new eligibility criteria parsing tool,^29^ which consists of a context-free grammar (CFG) and an information extraction (IE) modules to transform free-text eligibility criteria to structured formats for downstream analysis. The CFG module uses a lexer to divide criteria into tokens and a modified Cocke–Younger–Kasami algorithm to build parse trees from tokens, which are subsequently analyzed by removing duplicates and subtrees. The IE module uses an attention-based bidirectional long short-term memory with a conditional random field layer for named entity recognition to extract MeSH terms from criteria text. Based on the evaluation by Tseo et al,^29^ its performance is competitive in entity recognition, entity linking, and attribute linking. As it only extracted MeSH concepts from eligibility criteria but not their temporal constraints and other qualifiers, we called them “eligibility features” in this article. To evaluate its concept extraction accuracy, we manually reviewed a random sample of 300 rows of extracted results along with their original criteria. In the extracted results, there are cases where a term was identified but was not matched to a MeSH concept. In such cases, we considered them to be false negatives. The precision is 98.9%. The recall is 81.1%. The false negative ones were mostly quantitative criteria (29.3%) or due to missing concepts in MeSH (48.3%). We also identified the parsing errors of the frequent concepts and used Python to correct them in the whole parsing result. For example, we corrected the parsing results of the criterion “men”, which was parsed as “multiple endocrine neoplasia”. It is fine to miss some quantitative criteria as they were extracted by Valx^30^ with a high sensitivity and specificity. We also merged similar concepts in the parsing results based on the analysis needs. Detailed information about the merging of extracted concepts can be found in the Supplementary Material II. To evaluate the accuracy of extracting known risk factors from eligibility criteria using the tool,^29^ we reviewed a random sample of 300 rows of extracted risk factors; the precision is 100%. Regarding the recall, we took a random sample of 200 unique criteria. Seventy-two of them contain a risk factor and the program extracted 61 of them, making the recall to be 84.7%. After the categorical eligibility features of COVID-19 studies were parsed, we conducted 3 types of analyses: (1) frequency of the categorical eligibility features; (2) clustering analysis of the clinical studies based on the parsed eligibility features; and (3) frequency of exclusion eligibility features on chronic conditions and risk factors. Since (1) is intuitive, we explain the process of (2) and (3) in details as follows.

#### Clustering analysis of clinical studies

We used the clustering analysis to group the clinical studies based on their eligibility features. After the inclusion and exclusion criteria are parsed by the aforementioned tool,^29^ we utilized the parsed concepts as features to construct clinical study representation. For inclusion and exclusion eligibility features, we first removed the duplicated concepts for each clinical study. For example, if “pregnancy women” is mentioned multiple times in the exclusion criteria, only one was kept. Then, we appended the prefixes “inc” or “exc” to the concepts extracted from inclusion or exclusion criteria respectively to differentiate them. After data preprocessing, we constructed the data representations by treating each clinical study as a text document that contains concepts from inclusion and exclusion eligibility features. The Term Frequency-Inverse Document Frequency (TF-IDF) weighting scheme was employed to construct the feature vectors to feed to the K-means clustering algorithm.^31^ K-means is straightforward and can be applied to analyze large and high dimensional datasets. The algorithm assigns the instance to one of the clusters. The objective is to minimize the sum of the distances of the instances within the cluster to the cluster centroid. The silhouette value and CHindex were jointly used to measure the clustering results of K-means to determine the optimal number of clusters. The silhouette values measure similarity of an instance to its own cluster compared to other clusters. In this research, we experimented with k values from 2 to 50 for k-means. The optimal k was chosen when the silhouette value average of all instances is high and there are at least 20 instances for each cluster. To visualize the clustering result, we employed the uniform manifold approximation and projection (UMAP)^32^ to project the high dimensional data into 2-dimensional space for visualization. The UMAP reduces the dimensions by estimating the topology of the high dimensional data. It considers the local relationships within groups and global relationships between groups. UMAP can be applied directly to sparse matrices. In addition, we also clustered only the interventional studies considering both the extracted eligibility features and the enrollment values. The reason we included only interventional studies in this analysis is that observational studies often have a huge enrollment value, thereby dominating the clustering results. In addition, the eligibility criteria of observational studies are usually broad and unrestrictive. We used Principal Component Analysis (PCA) to reduce the dimensionality of eligibility features (weighted by TF-IDF) to k and then added 2 more features: the enrollment value (normalized to 0-1) and the intervention type, due to their importance deemed by the study team. The original dimension is 2608 presenting the total number of eligibility features. Since we add 2 study features, in order to avoid biases and preserve the data distribution on eligibility features, we experimented with k value from 5 to 15. There is no significant difference between the clustering results based on the optimal silhouette and the cluster distribution. Hence, k value was set to 9 in this study.

#### Exclusion eligibility features on chronic conditions and risk factors

First, we examined the upper limit and lower limit of the age criterion, which are structured data in the study summaries. Then, from the results of the criteria parsing tool,^29^ we examined the use of exclusion eligibility features for 15 most prevalent chronic conditions among older adults in the National Inpatient Sample of the Healthcare Cost and Utilization Project (HCUP) (appearing in over 6% of the older adults in NIS).^33^ These conditions include hypertension, hyperlipidemia, ischemic heart disease, diabetes, anemia, chronic kidney disease, atrial fibrillation, heart failure, chronic obstructive pulmonary disease and bronchiectasis, rheumatoid arthritis or osteoarthritis, acquired hypothyroidism, Alzheimer disease and related disorders or senile dementia, depression, osteoporosis, and asthma. In addition, we also considered 3 chronic conditions that are prevalent in all adults: cancer, stroke, and high cholesterol. We then analyzed the use of risk factors that may lead to serious illnesses in the eligibility criteria.

All the data and codes pertaining to this project have been deposited to GitHub: https://github.com/ctgatecci/Covid19-clinical-trials-11-27-2020.

## RESULTS

### Basic characteristics of the COVID-19 clinical studies

**Figure 1.**
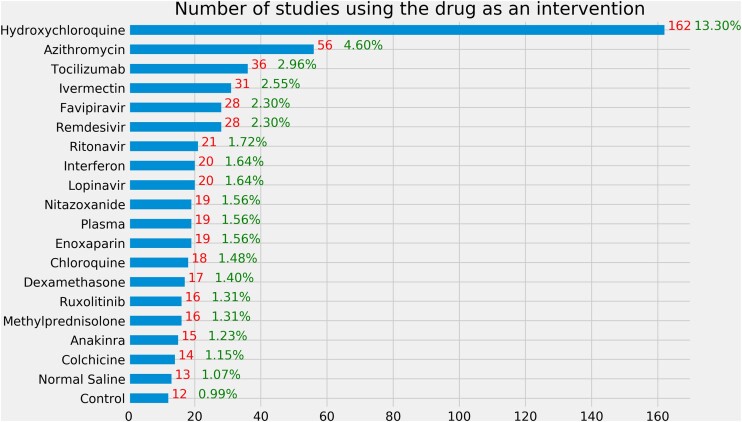
Number of interventional studies using a drug as an intervention. The denominator is the 1218 interventional studies using drug as an intervention. Note that some studies tested multiple drugs.

Table 2 shows the basic characteristics of 3765 COVID-19 clinical studies in ClinicalTrials.gov. Among 3765 clinical studies included in this article, a majority of them are interventional studies (clinical trials). Among those interventional studies, 43.4% are in Phase 2 or 3. Most of the studies (83.19%) are sponsored by hospitals, universities, research institutes, or individuals. Besides drugs, other interventions include biological (15.9%), behaviors (7.06%), device (6.01%), diagnostic test (3.57%), and others (10.72%, eg, genetic, dietary supplements, radiation, and combination). The majority of the studies focused on treatment (40.88%) and prevention (8.95%). The 10 most frequently tested drugs in different clinical studies are hydroxychloroquine (*N* = 162), azithromycin (*N* = 56), tocilizumab (*N* = 36), ivermectin (*N* = 31), favipiravir (*N* = 28), remdesivir (*N* = 28), ritonavir (*N* = 21), lopinavir (*N* = 20), interferon (*N* = 20), and plasma (*N* = 19) (Figure 1).

**Table 2.** Basic characteristics of 3765 COVID-19 clinical studies in ClinicalTrials.gov

Characteristics	Number of studies	Percentage
Study type		
Interventional	2295	60.95
Observational	1470	39.05
Study phase (Interventional study only)		
Phase 2	559	24.36
Phase 3	350	15.25
Phase 1	171	7.45
Phase 2/Phase 3	156	6.80
Phase 1/Phase 2	138	6.01
Phase 4	108	4.71
Early Phase 1	38	1.66
N/A	775	33.77
Gender		
Female only	69	1.83
Male only	29	0.77
Both	3677	97.40
Overall status		
Recruiting	1910	50.73
Not yet recruiting	851	22.60
Completed	479	12.72
Active, not recruiting	280	7.44
Enrolling by invitation	121	3.21
Withdrawn	64	1.70
Terminated	34	0.90
Suspended	26	0.69
Sponsor		
Industry	578	15.35
NIH	46	1.22
US federal agencies	9	0.24
Other^a^	3132	83.19
Intervention type (interventional studies only)		
Drug	1156	50.37
Biological	365	15.90
Other^b^	246	10.72
Behavioral	162	7.06
Device	138	6.01
Diagnostic test	82	3.57
Dietary supplement	62	2.70
Procedure	44	1.92
Combination product	23	1.00
Radiation	16	0.70
Genetic	1	0.04
Primary purpose		
Treatment	1539	40.88
Prevention	337	8.95
Other	110	2.92
Supportive care	106	2.82
Diagnostic	101	2.68
Health services research	47	1.25
Basic science	26	0.69
Screening	22	0.58
Device feasibility	7	0.19
N/A	1470	39.04
Allocation (interventional studies only)		
Randomized	1688	73.55
Non-randomized	191	8.32
N/A	416	18.13
Intervention model		
Parallel assignment	1632	43.35
Single group assignment	450	11.95
Sequential assignment	122	3.24
Crossover assignment	57	1.51
Factorial assignment	34	0.90
N/A	1470	39.04

*Note*: ^a^“Other” includes hospitals, universities, research institutes, and individuals.

b “Other” includes dietary supplements, genetic, radiation, and combination product.

### Risk factors in trial description

**Figure 2.**
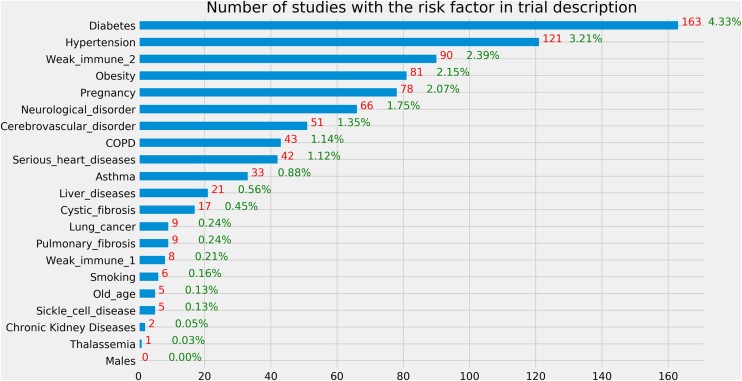
Number of studies with a risk factor for severe illness in the trial description. The denominator is the 3765 clinical studies included in this study.

Figure 2 illustrates the occurrences of the risk factors in the study description of the included studies. We merged the brief summary and detailed description. “Weak immune 1” corresponds to immunocompromised state from solid organ transplant and “weak immune 2” corresponds to immunocompromised state from blood or bone marrow transplant, immune deficiencies, HIV, use of corticosteroids, or use of other immune-weakening medicines. The top 5 risk factors mentioned in trial description are diabetes, hypertension, weak immune 2, obesity, and pregnancy. According to the *t*-test result, on average, interventional studies mentioned fewer risk factors in trial description than observational studies (mean value: 0.2 vs 0.27, *P *=* *.002, 2-tailed *t*-test). The number of risk factors mentioned in trial description was significantly associated with the intervention type (*P* < .001, ANOVA), while its association with the primary purpose was not statistically significant (*P* = .078, ANOVA).

### Quantitative criteria

**Figure 3.**
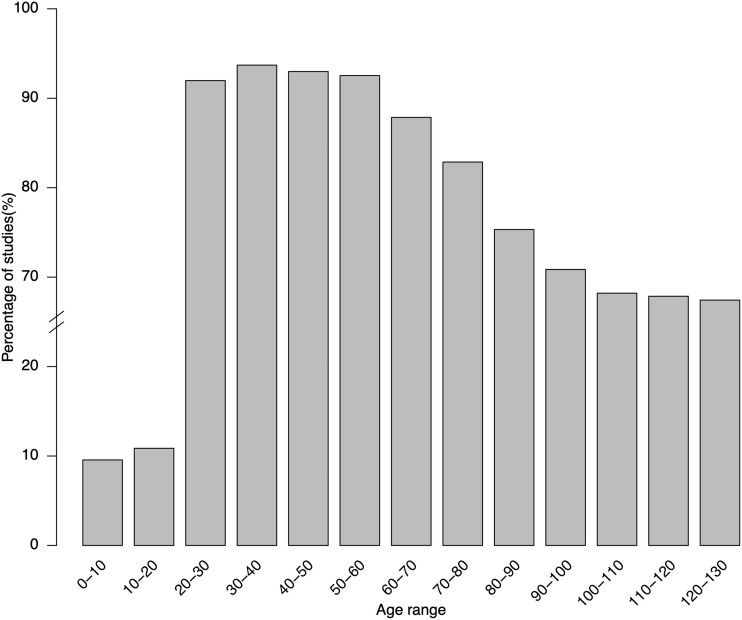
Percentage of COVID-19 clinical studies allowing age ranges.

Table 3 lists the top 20 frequently used quantitative criteria in COVID-19 clinical studies. Note that the “age” criterion is also a structured field in the study records. Based on the analysis of upper age limit, 67.3% (*N* = 2534) clinical studies do not have an upper age limit. For those that have an upper age limit, the most frequent limits are 80 (*N* = 191), 75 (*N* = 117), 65 (*N* = 108), 100 (*N* = 99), and 70 (*N* = 94). Regarding the lower age limit, only 9.8% studies (*N* = 369) do not have a lower age limit. Most frequently used lower age limits are 18 (*N* = 2856), 16 (*N* = 59), 20 (*N* = 49), 19 (*N* = 32), and 50 (*N* = 31). Figure 3 illustrates the percentage of COVID-19 clinical studies that consider each age range. In general, patients who are over 18 years old are considered while those over 70 years old are less considered than 18–70 years old. Regarding oxygen saturation, most studies use 93% (*N* = 114), 94% (*N* = 58), or 90% (*N* = 29) as threshold values.

**Table 3.** Top 20 frequently used quantitative criteria in COVID-19 clinical studies

Rank	Criteria	Frequency	Percentage	Rank	Criteria	Frequency	Percentage
1	Age	2255	70.4	11	Platelet count	108	3.4
2	Oxygen saturation	229	7.2	12	ANC	106	3.3
3	Pao2/fio2	223	7.0	13	Creatinine clearance	105	3.3
4	BMI	222	6.9	14	Heart rate	75	2.3
5	Respiratory rate	190	6.0	15	Diastolic blood pressure	64	2.0
6	AST	185	5.8	16	QTC	63	2.0
7	EGFR	145	4.5	17	Total bilirubin level	56	1.8
8	Temperature	131	4.1	18	Pulse rate	52	1.6
9	Systolic blood pressure	113	3.5	19	Hemoglobin	48	1.5
10	ALT	109	3.4	20	Creatinine	46	1.4

### Categorical eligibility features

**Figure 4.**
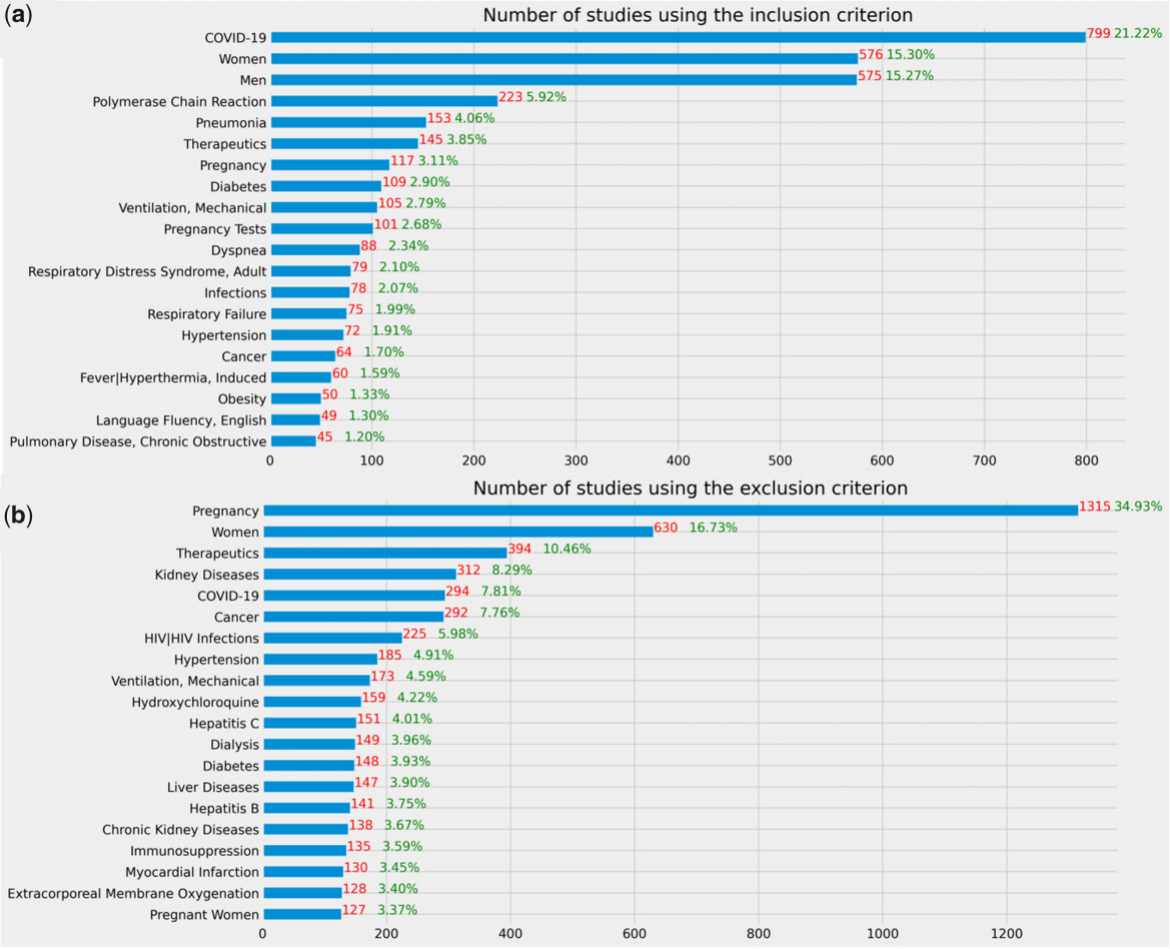
Frequent eligibility features of COVID-19 clinical studies. The denominator is the 3765 clinical studies included in this study.

Figure 4 illustrates frequent concepts extracted from inclusion and exclusion criteria of COVID-19 clinical studies. According to these results, COVID-19 diagnosis, polymerase chain reaction, pneumonia, diabetes, therapeutics, mechanical ventilation were often used eligibility features in the inclusion criteria, whereas pregnancy, therapeutics, kidney diseases, cancer, HIV, mechanical ventilation, hydroxychloroquine, and hepatitis C were often used eligibility features in the exclusion criteria.

**Figure 5.**
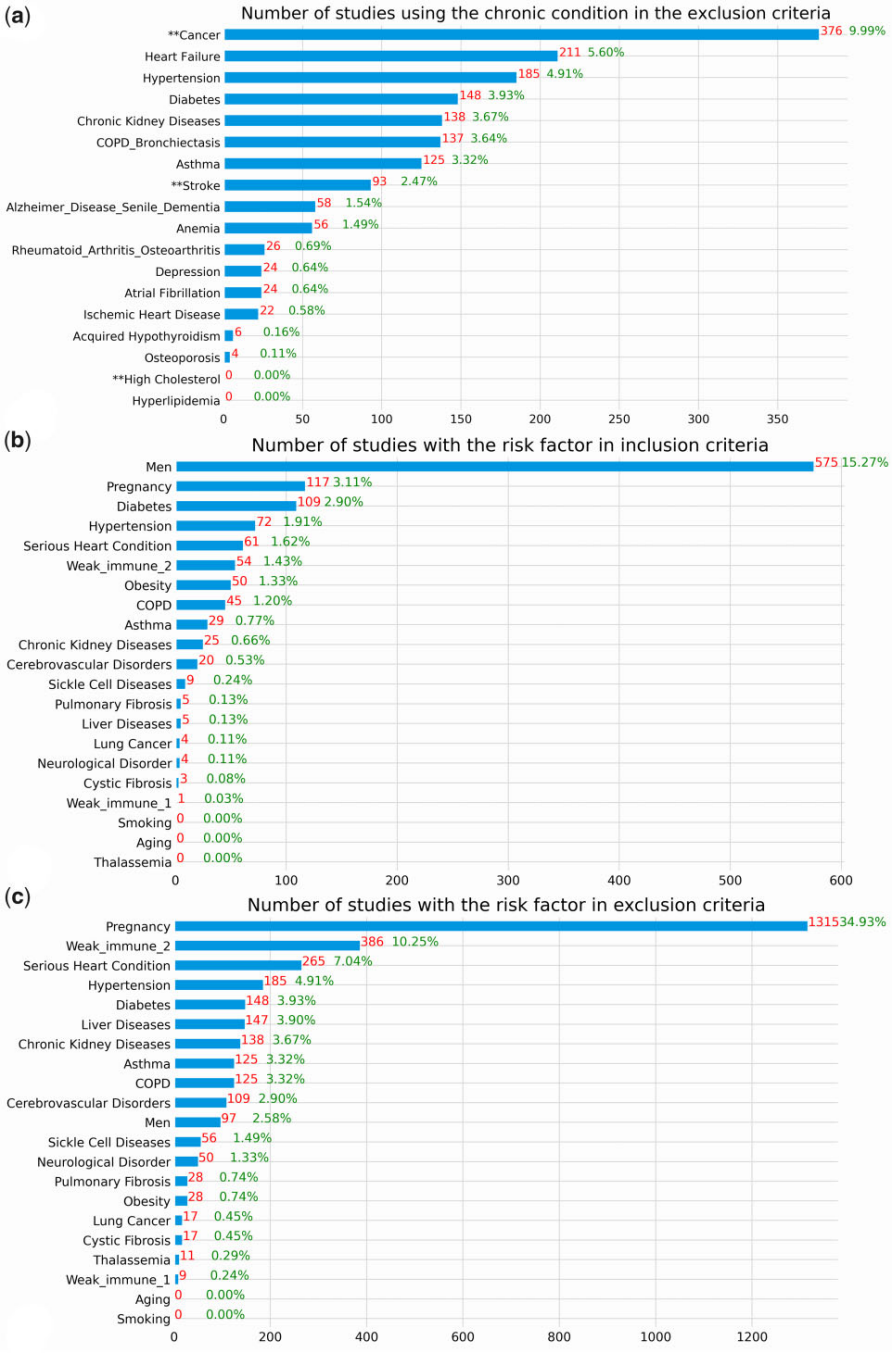
(A) Number of studies using a prevalent chronic condition among the older adults in exclusion criteria. **Represents the conditions that are not in the list of top 15 prevalent conditions among older adults but prevalent in younger adults. (B) Number of studies with the risk factor in inclusion criteria (C) Number of studies with the risk factor in exclusion criteria. The denominator of these 3 figures is the 3765 clinical studies included in this study.

Figure 5 shows the number of studies that used an exclusion eligibility feature about a common chronic condition prevalent among older adults in the included studies as well as the risk factors for serious illnesses in the eligibility criteria. Even though a majority of studies did not exclude patients with these chronic conditions, some highly prevalent chronic conditions such as cancer, heart failure, hypertension, diabetes, chronic kidney disease, and COPD are among the most frequently used exclusion criteria in 3.64%–9.99% studies. Few studies purposely included patients with a risk factor that may lead to serious illnesses, but many studies explicitly excluded them, especially pregnant women. According to the results of the statistical tests, on average, interventional studies used more risk factors in eligibility criteria than observational studies (mean: 1.19 vs 0.22, *P* < .001, 2-tailed *t*-test). There is a statistically significant association between the number of risk factors used in eligibility criteria and the intervention type (*P* < .001, ANOVA), and primary purpose of the studies (*P* < .001, ANOVA).

**Figure 6.**
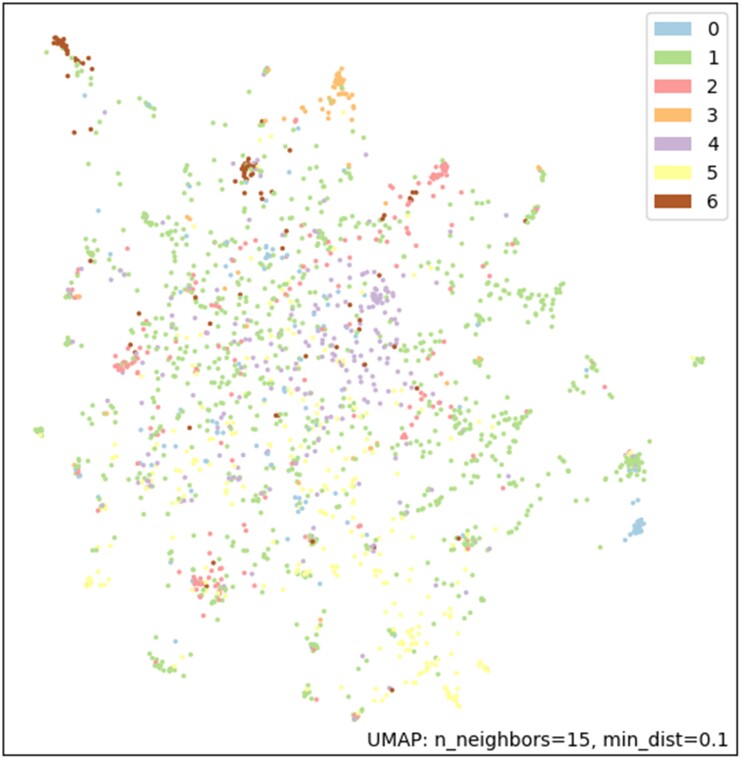
Visualization of the 7 clusters using uniform manifold approximation and projection.

Table 4 shows the top 10 frequent inclusion and exclusion features used in the studies in each of the 7 clusters resulting from the clustering analysis with eligibility features only. Pregnancy is the most frequent exclusion criterion in all the 7 clusters. Clusters #0 and #1 are the largest clusters with low silhouette value and the trials in these clusters often excluded patients with different diseases. Studies in Cluster #0 often included patients with pneumonia and excluded patients with cognition/cognitive behavioral therapy/cognitive dysfunction. Studies in Cluster #1 often excluded patients who are on therapeutics, kidney diseases, and cancer. Studies in Cluster #2 often included patients with polymerase chain reaction. All the trials in Cluster #3 excluded pregnant women. Studies in Cluster #5 often excluded HIV/HIV Infections, Hepatitis B, Hepatitis C, and Cancer. Cluster #6 has the highest silhouette value which indicates that the studies in it are more cohesive than those in other clusters. Most studies in Cluster #6 have no exclusion criteria. Through the clustering analysis, we can help classify studies on relationships not available a priori and identify sets of clinical studies focusing on different study population. Figure 6 shows the visualization of these 7 clusters using UMAP. The detailed results of the clustering analysis of the COVID-19 clinical studies are provided in the Supplementary Material III. The results of the clustering analysis of the interventional studies when considering the eligibility features, the enrollment, and the intervention type are provided in the Supplementary Material IV (table and figure) and V (detailed results).

**Table 4.** Top 10 frequently used concepts in inclusion criteria and exclusion criteria of the studies in each cluster of the clustering analysis with eligibility features

Cluster number	Number of studies	Total enrollment	Silhouette scores	Inclusion criteria	Exclusion criteria
0	124	38 351	0.0112027	Pneumonia (*N* = 90), COVID-19 (*N* = 49), Women (*N* = 21), Men (*N* = 19)	Pregnancy (*N* = 77), cognition/cognitive behavioral therapy/cognitive dysfunction (*N* = 35), women (*N* = 30), therapeutics (*N* = 19), HIV/HIV infection (*N* = 16), kidney diseases (*N* = 15)
1	975	853 187	−0.019703	COVID-19 (*N* = 199), Women (*N* = 101), Men (*N* = 86),	Pregnancy (*N* = 427), women (*N* = 160), therapeutics (*N* = 149), kidney diseases (*N* = 134), Cancer (*N* = 100), hypertension (*N* = 79), liver diseases (*N* = 79)
2	216	71 812	0.0103414	Polymerase chain reaction (*N* = 52), COVID-19 (*N* = 37), diabetes (*N* = 36), hypertension (N = 22), obesity (*N* = 19)	Pregnancy (*N* = 198), women (*N* = 124), therapeutics (*N* = 23), pregnancy tests (*N* = 20), hydroxychloroquine (*N* = 19)
3	73	16 039	0.0848274	COVID-19 (*N* = 18), women (*N* = 7), men (*N* = 6)	Pregnant women (*N* = 73), women (*N* = 15), cancer (*N* = 10), asthma (*N* = 7), kidney diseases (*N* = 6), diabetes (*N* = 6), heart failure (*N* = 6)
4	252	107 769	0.0076462	Men (*N* = 232), women (*N* = 219), COVID-19 (*N* = 72), pregnancy tests (*N* = 37)	Pregnancy (*N* = 165), women (*N* = 90), therapeutics (*N* = 61), dialysis (*N* = 38), ventilation mechanical (*N* = 36), COVID-19 (*N* = 30)
5	297	502 661	0.0052849	Men (*N* = 128), women (*N* = 121)	Pregnancy (*N* = 221), HIV/HIV infections (*N* = 159). Women (*N* = 125), hepatitis B (*N* = 109), hepatitis C (*N* = 104), cancer (*N* = 101), therapeutics (*N* = 97), COVID-19 (*N* = 81)
6	105	103 814	0.2595492	COVID-19 (*N* = 96), women (*N* = 7), men (*N* = 5)	Pregnancy (*N* = 33), COVID-19 (*N* = 30), women (*N* = 14), therapeutics (*N* = 7), cancer (*N* = 5), kidney diseases (*N* = 4), therapies investigational (*N* = 4)

## DISCUSSION

As the novel coronavirus COVID-19 has significantly impacted our lives and even taken lives of almost 3 millions of people so far, we must quickly identify repurposed drugs or develop new drugs and vaccines to safely and effectively control the spread of the virus and save lives. Clinical studies, especially RCTs, are a fundamental tool used to evaluate the efficacy and safety of new medical interventions for disease prevention or treatment. Many clinical studies are being conducted to find safe and effective treatments and vaccines. Thus far, significant efforts have been devoted to repurposing existing FDA-approved drugs including immunosuppression (eg, hydroxychloroquine, tocilizumab), antivirus (eg, fevipiravir, lopinavir/ritonavir), antiparasite (eg, ivermectin, nitazoxanide), antibiotics (eg, azithromycin), and anticoagulant (eg, enoxaparin). In our analysis of the COVID-19 clinical studies, we found that the use of eligibility criteria and consideration of risk factors in these studies did not change much from June 18, 2020 to November 27, 2020 even though the number of COVID-19 studies in ClinicalTrials.gov grew from 2192 to 4028.

To transform clinical trials and lower their cost, a notion of “digital clinical trial” was created to leverage digital technology to improve important aspects such as patient access, engagement, and trial measurement.^34^ The US National Institutes of Health and the National Science Foundation held a workshop in April 2019 about the implementation of digital technologies in clinical trials, in which “defining and outlining the composition and elements of digital trials” and “elucidating digital analytics and data science approaches” were identified as 2 of the 5 top priorities. As COVID-19 is a major health crisis that impacts people regardless of their age, gender, and race/ethnicity, it is in our interest to understand if clinical studies on COVID-19 adequately considered the representation of real-world populations. Based on our analysis, most clinical studies consider both genders (97.4%, *N* = 3667), do not have an upper age limit 67.3% (*N* = 2534), and have a lower age limit of 18 (75.9%, *N* = 2856). The exclusion of children in these studies may be due to lower susceptibility and lower rates of mortality and hospitalization for children with COVID-19 compared to adults.^35^ As serious illnesses of COVID-19 mostly occurred in older adults with underlying health conditions, it is not surprising that they are in general considered by most COVID-19 studies, based on our analysis of their eligibility criteria. Most studies did not set an upper age limit (67.3%, *N* = 2534) and did not exclude older adults with common chronic conditions. This is contrary to the recent New York Times articles conjecturing that older people are left out form COVID-19 trials.^10^ As older adults are the most likely to be hospitalized due to COVID-19, clinicians may be more likely to choose to include them to fulfill the sample size requirement of the trials. Nonetheless, conducting COVID-19 clinical studies could still be challenging in the traditional clinical trial eco-system, where patient accrual is often delayed due to logistical constraints.^36^ The generalizability of the study results to the real-world population should be evaluated with state-of-the-art techniques.^9^ Older adults could have still been underrepresented in COVID-19 clinical studies due to logistical reasons, which can only be assessed with the published results after the completion of the studies.^37^ In addition, pregnant women are often excluded in COVID-19 studies. Even though pregnant women are in general excluded in most clinical trials due to the potential risks to both the women and the unborn babies, observational studies should carefully evaluate the vertical transmission of the virus and negative impact of COVID-19 on the well-being of mothers and infants.^38^ Recently, Director of the National Institute of Child Health and Human Development published a viewpoint article in JAMA to call for greater inclusion of pregnant and lactating women in COVID-19 vaccine clinical research.^39^ Clinical studies should adequately evaluate the efficacy and safety of treatments and vaccines on vulnerable population groups.

### Limitations

A few limitations should be noted. First, some data in ClinicalTrials.gov are missing. For example, 33.8% (*N* = 775) of the interventional studies miss study phase information. 39% (*N* = 1470) of studies do not have primary purpose information. Second, we relied on the search function of ClinicalTrials.gov when retrieving COVID-19 studies. There may be study indexing errors, but the scale should be minimal and would not impact the findings. Third, we used the QuickUMLS and the new eligibility criteria parsing tool^29^ to extract risk factors, chronic conditions, disorders, and procedures from study records. Thus, the sensitivity and specificity of the term extraction and normalization are dependent on the quality of the UMLS Metathesaurus and the eligibility criteria parsing tool. Nonetheless, we have carefully curated the term extraction results to ensure that our results are as accurate as possible.

## CONCLUSIONS AND FUTURE WORK

In this article, we systematically analyzed COVID-19 clinical study summaries in ClinicalTrials.gov using NLP. Specifically, we analyzed whether these clinical studies considered the underlying health conditions (and other risk factors) that may increase the severity of the COVID-19 illness. Given the ongoing nature of this pandemic, it is inevitable that early trials will start with different knowledge of risk factors than later trials. In future work, we will perform a longitudinal analysis of COVID-19 studies to assess the changes in the use of eligibility criteria and consideration of risk factors for severe illness in COVID-19 patients. As results of COVID-19 studies become available, we will be able to assess the extent to which the trial design and eligibility criteria in particular would impact the findings as well as the real-world population representativeness of these studies using generalizability assessment methods.^9^

## DATA AVAILABILITY

The data underlying this article were accessed from ClinicalTrials.gov. The derived data and code generated in this research are publicly available at https://github.com/ctgatecci/Covid19-clinical-trials-11-27-2020.

## FUNDING

This study was partially supported by the National Institute on Aging (NIA) of the National Institutes of Health (NIH) under Award Number R21AG061431; and in part by Florida State University-University of Florida Clinical and Translational Science Award funded by National Center for Advancing Translational Sciences under Award Number UL1TR001427. The development of Valx was supported by National Library of Medicine Grant R01LM009886. The content is solely the responsibility of the authors and does not necessarily represent the official views of the NIH.

## AUTHOR CONTRIBUTIONS

Z.H. conceived, designed, guided, and coordinated the study and the writing. Z.H. collected the data from ClinicalTrials.gov, performed the data analyses, interpreted the results, and drafted the manuscript. A.E. performed the NLP of the clinical study records. X.L. performed the clustering analysis. A.X. performed statistical tests to assess the association between the occurrences of risk factors and the study characteristics. All the authors edited the manuscript thoroughly. The submitted manuscript has been approved by all the authors.

## SUPPLEMENTARY MATERIAL

Supplementary material is available at *Journal of the American Medical Informatics Association* online.

## Supplementary Material

ooab032_Supplementary_Data
