# Supplementary material for: How the clinical research community responded to the COVID-19 pandemic: an analysis of the COVID-19 clinical studies in ClinicalTrials.gov
Source: JAMIA Open. 2021 Apr 20;4(2):ooab032. doi: 10.1093/jamiaopen/ooab032 (PMC8083215; doi:10.1093/jamiaopen/ooab032)
Supplement: ooab032_Supplementary_Data [file ooab032_supplementary_data.zip › Supplementary_Material_IV.pdf]

Appendix Table 1. Top 10 frequently used concepts in inclusion criteria and exclusion criteria of the studies in each cluster of the clustering analysis with eligibility features, intervention type, and enrollment.

Cluster Number	Number of Studies	Total Enrollment	Silhouette scores	Intervention Type	Inclusion Criteria	Exclusion Criteria
0	120	96834	0.1652518	Drug (117), Dietary (2), Supplement (2), Other (1)	COVID-19 (36), Men (27), Women (27)	Hydroxychloroquine (114), Pregnancy (79), Chloroquine (79), Women (46), Therapeutics (33), Glucosephosphate dehydrogenase deficiency (32), Azithromycin (31)
1	532	667023	0.4460913	Biological (329), Behavioral (95), Device (87), Product (21), Combination (21)	Women (139), Men (134), COVID-19 (113),	Pregnancy (259), Women (133), Cancer (94), Therapeutics (92), COVID-19 (85), HIV/HIV infections (77), Hypertension (67)
2	135	52626	0.006419	Drug (100), Other (13), Diagnostic (9), Test (9), Dietary (5), Supplement (5), Device (4), Procedure (2), Radiation (1), Behavioral (1)	Men (130), Women (119), COVID-19 (22),	Pregnancy (83), Women (41), Therapeutics (25), Cancer (15), Kidney diseases (15), COVID-19 (14), Dialysis (13)
3	23	12864	0.3585205	Drug (11), Other (5), Diagnostic (2), Test (2), Biological (2), Device (1), Procedure (1), Dietary (1), Supplement (1)	Polymerase chain reaction (6)	Pregnancy (23), Women (12), COVID-19 (1)
4	833	694010	0.3420431	Drug (611), Other (129), Dietary (35), Supplement (35), Procedure (24), Diagnostic (23), Test (23), Radiation (11)	Women (137), Men (123), Polymerase chain reaction (78)	Pregnancy (480), Women (225), Therapeutics (146), Kidney diseases (130), Cancer (103), Ventilation mechanical (82), HIV/HIV infections (77)
5	52	32242	0.2909869	Drug (13), Biological (10), Device (9),	COVID-19 (52), Women (3), Men (3),	Pregnancy (15), Women (6), COVID-19 (4), Cancer (1)

				Diagnostic (7), Test (7). Other (7), Behavioral (3), Procedure (2), Dietary (1), Supplement (1)	Polymerase chain reaction (1), Respiratory failure (1), Diabetes (1)	
6	33	9378	0.2354992	Drug (8), Device (8), Behavioral (8), Other (7), Biological (1), Dietary (1). Supplement (1)	COVID-19 (8), Women (4)	Cognition/Cognitive behavioral therapy/Cognitive dysfunction (33), Pregnancy (13), Hypertension (7), Women (5), Therapeutics (5), Kidney diseases (4), Cancer (4), Immunosuppresive agents (3)
7	49	8874	0.0995203	Drug (16), Other (8), Diagnostic (6), Test (6), Device (6), Biological (6), Dietary (5), Supplement (5), Procedure (2)	COVID-19 (9), Men (5), Women (5), Pneumonia (3), Polymerase chain reaction (3)	Pregnant women (49), Women (9), Cancer (4), Asthma (4), Diabetes (3)
8	265	119782	0.1314706	Drug (215), Other (26), Dietary (8), Supplement (8), Procedure (8), Radiation (4), Diagnostic (3), Test (3), Device (1)	COVID-19 (256), Men (57), Women (53), Pneumonia (27)	Pregnancy (169), Women (81), Therapeutics (55), COVID-19 (40), Kidney diseases (35), Ventilation mechanical (34)

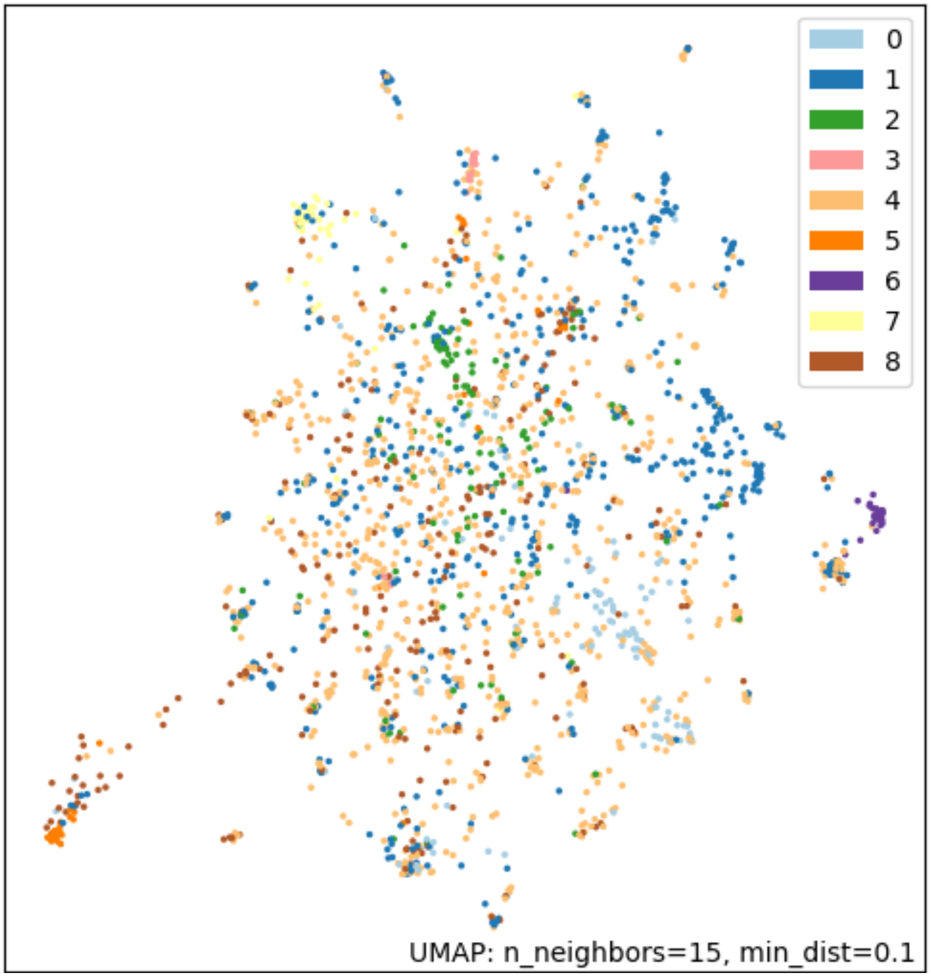

Appendix Figure 1. Visualization of the 9 clusters using UMAP
